# Randomized Controlled Trial Outcomes for HomeStyles-2, an Online Obesity Prevention Program for Families with Children in Middle Childhood

**DOI:** 10.3390/nu18071029

**Published:** 2026-03-24

**Authors:** Carol Byrd-Bredbenner, Angelica A. Pozzoli, Kaitlyn M. Eck, John Worobey, Karla Pagan Shelnutt, Melissa D. Olfert, Virginia Quick

**Affiliations:** 1 Nutritional Sciences Department, Rutgers University, New Brunswick, NJ 08901, USA; aap227@sebs.rutgers.edu (A.A.P.); worobey@sebs.rutgers.edu (J.W.); vquick@njaes.rutgers.edu (V.Q.); 2 Department of Nutritional Sciences, Thomas Jefferson University, Philadelphia, PA 19144, USA; kaitlyn.eck@jefferson.edu; 3 Department of Family, Youth, and Community Sciences, University of Florida, Gainesville, FL 32611, USA; kpagan@ufl.edu; 4 School of Agriculture and Food Systems, West Virginia University, Morgantown, WV 26506, USA; melissa.olfert@mail.wvu.edu

**Keywords:** nutrition, health, middle childhood, parents, lifestyles, intervention, obesity, cognitions, home environment, social cognitive theory

## Abstract

**Background:** Parents are children’s primary role models, are food and physical activity gatekeepers, and create the home structure/lifestyle environment. Thus, parents strongly influence children’s weight-related behaviors and have the opportunity to cultivate a “culture of health” within the home. **Methods:** The aim of the HomeStyles-2 (also called HomeStyles-Child) RCT was to determine whether this online, novel, age-appropriate, family intervention enabled and motivated the 131 systematically randomly assigned by computer parents of children in middle childhood (ages 6 to 11) in the experimental condition to shape home environments and healthy weight-related lifestyle practices to be more supportive of optimal health and reduced obesity risk in middle childhood youth more than the 134 counterparts assigned to the attention control condition. **Results:** This RCT demonstrated the feasibility of online delivery of a health promotion intervention to parents of children in middle childhood, which may inform the development of interventions targeting other age groups and health outcomes. Results indicate the HomeStyles-Child intervention improved healthy-weight-related behavior cognitions, which are predictors of behavior change, of the experimental group. Additionally, improvements in experimental parent and child health-related behaviors were observed. These improvements occurred during a time when families faced unprecedented and extraordinary economic and social stresses associated with the COVID-19 pandemic. **Conclusions:** HomeStyles-Child is one of the few interventions for families with middle childhood youth. It has the potential to help ameliorate obesity in middle childhood youth and, by extension, other family members.

## 1. Introduction

The prevalence of obesity in school-age children (ages 6 to 11 years) living in the United States is 20.7% [1], which is concerning given that children with obesity face greater physical and mental health risks than their peers at healthy weights. For example, children with obesity experience increased risk for chronic disease, including cardiovascular disease, type 2 diabetes mellitus, metabolic dysfunction-associated steatotic liver disease, and metabolic dysfunction-associated steatohepatitis [2,3,4,5,6]. Obesity-related chronic disease tends to track into adulthood and result in reduced quality of life, shorter life expectancy, and greater healthcare needs and associated costs [2,7,8,9,10,11,12]. Children with obesity also face mental health risks, including depression and anxiety disorders, often exacerbated by weight-based bullying and stigmatization [13,14,15,16].

As children’s role models and household gatekeepers, parents play a pivotal role in promoting family health and creating a “culture of health” within the home [7,17,18,19,20,21,22,23,24,25,26,27,28,29,30,31,32,33,34,35,36,37,38,39,40]. Evidence indicates that important predictors of child physical activity levels are parent attitudes towards physical activity, behavioral modeling of physical activity, and encouragement of children to be active [22,23]. Similarly, parent consumption of healthy foods is positively correlated with their children’s dietary intake [25,26,28,29,41]. By establishing healthy lifestyle practices within the home environment, such as mealtime patterns, family activity habits, and sleep hygiene practices, parents have the opportunity to create environments that ameliorate obesity risk factors in lifestyle practices (i.e., diet, exercise, sleep) and home environments [22,27,30,31,32,42,43,44,45,46,47,48,49,50,51,52,53,54,55,56]. Despite the myriad benefits of educating parents, few research-based obesity prevention programs are available, particularly for families with school-age children [19,57,58,59].

School-age youth are in a life stage commonly called “middle childhood” [60,61]. During this physically, socially, cognitively, and emotionally distinct life stage [60,62,63], bounded by the preschool years on one end and adolescence at the other, children’s cognitive capacities rapidly develop. Socially, they become more independent, and their networks grow to include many others beyond the family [60,61]. Children in middle childhood become more self-aware and are able to plan ahead, assess and adjust their actions to progress toward goals while gaining self-confidence and pride in their abilities to be productive and contribute to group enterprises [60,61]. The developmental tasks of this life stage present an opportune time for parents to assist their children in gaining health-promoting knowledge and skills that will help them now and later as they pass into adolescent and adult years.

The HomeStyles-2, also called HomeStyles-Child, intervention was developed in direct response to the dearth of obesity prevention programs available for families with children in middle childhood. This intervention was created and evaluated using methods analogous to those applied to HomeStyle-1, also called HomeStyles-Pre, which was designed for families with preschool children [64,65]. In brief, HomeStyles is multi-faceted because dietary, physical activity, and sleep behaviors are linked with obesity risk, and addressing all of the facets improves the likelihood of positive outcomes [66,67,68,69]. HomeStyles is also congruent with research-based obesity prevention recommendations from leading authorities (e.g., Institute of Medicine, Centers for Disease Control and Prevention, Healthy People 2030 goals, Dietary Guidelines for Americans) [19,70,71,72,73,74,75,76,77,78,79]. In the development of the intervention and assessment tools, HomeStyles applied community-engaged research principles through intensive consultation with the target audience (i.e., parents and children) [80,81,82]. HomeStyles is predicated on a social ecological framework that situates individuals within the context of their home physical and social environments and draws on Bandura’s reciprocal determinism notion that individuals and their environments simultaneously and mutually affect each other [83,84,85,86]. Home is the focus of HomeStyles because of the central and influential role of this environment on family health, and further elucidation of the impact of this environment will be valuable to health promotion efforts [85,87,88,89,90]. In addition, evidence from the limited available studies suggests that reshaping the home environment has many benefits, including reducing obesity risk [65,85,87,88,89,91,92,93].

There are two delivery modes for HomeStyles-Child: independent online learning and synchronous SNAP-Ed educational sessions [86,94]. The aim of this manuscript is to describe the outcomes of the randomized controlled trial (RCT) of the independent online electronic learning mode between baseline and post-intervention data collection. The goal of the RCT was to determine whether, at post-intervention, parents in the experimental group shaped their home environments and weight-related lifestyle practices to be more supportive of optimal health and reduced risk of obesity in their middle childhood youth than those in the control group.

## 2. Materials and Methods

This RCT was registered at clinicaltrials.gov, ID NCT04802291, on 14 March 2021, https://clinicaltrials.gov/study/NCT04802291; the study results are not yet publicly posted. The institutional review board approved this minimal risk behavioral intervention study (Rutgers University, PRO#2020001192), which included an experimental group and an attention control group. All participants gave informed consent online as a requirement for enrollment. Study methods were described in detail in the published protocol for this study [86] and are summarized below, along with deviations necessitated by the COVID-19 pandemic.

### 2.1. Sample Eligibility Criteria and Recruitment

As indicated in the protocol for this study, eligibility criteria were: being a parent between the ages of 24 and 50 years with at least 1 child aged 6 to 11 years, being the primary food gatekeeper in the household (i.e., makes all or most decisions related to family food choices), having regular Internet access, ability to read English and/or Spanish, and residing in the United States. A professional study recruitment company conducted all study participant recruitment activities. Due to extensive delays caused by COVID-19 quarantine restrictions, the profound effects of the pandemic on parents (e.g., working from home while simultaneously home-schooling their children, which diminished their interest in and availability for engaging in the intervention activities), attrition of key study personnel (e.g., student assistants who graduated and took employment elsewhere), and dwindling research funding (e.g., grant-funded study personnel employment was necessarily sustained during the COVID-19 quarantine), the eligibility criteria were modified to better ensure an adequate sample size by more sharply targeting recruitment efforts. Specifically, eligibility was limited to mothers (note: this decision was informed by the low participation and completion rate of fathers vs. mothers in HomeStyles-Pre) [65]. The English and Spanish interventions had the same components and were planned as separate analyses; however, due to financial constraints of grant funding caused by the lengthy COVID-19 pandemic and the lack of Spanish-speaking participants in the professional survey recruitment company database (note: originally, the company was only going to assist and not be the only recruitment activity), recruitment for the Spanish intervention was not attempted.

G*Power software version 3.1.9.2 (Universität Kiel, Kiel, Germany), calculated *a priori* for repeated measures analysis of variance with an effect size of 0.25, *p* < 0.05, and 80% power, indicated the sample size goal was 269 (half in each treatment group) [95,96]. The participant recruitment goal was set at a rate of 40% higher due to the length of the intervention and environmental pressures typically reported by parents [97,98]. Other *a priori* actions known to facilitate recruitment and retain participants were taken, including providing modest stipends to participants after completing each data collection point (i.e., baseline, post, follow-up), delivering the intervention electronically, thereby making access convenient, weekly mailings of intervention support materials designed for school-age children to participant homes, sending SMS (short message service) encouraging messages (nudges) several times weekly [99,100], easy access to project staff for troubleshooting, and blinding participants to treatment group assignment using recruitment materials applicable to both treatments along with providing a bona fide, credible, structurally equivalent attention control group treatment (i.e., equivalent in delivery format, attractiveness, value, and time commitment to the experimental treatment) [101,102,103,104,105,106,107].

Study recruitment announcements were electronically distributed by the study recruiter to potentially eligible participants. The announcements invited participants to sign up for an online program to learn simple steps that help kids grow up even happier, healthier, and safer. Interested participants completed an online screener survey to assess eligibility criteria, and if eligible, were presented with the informed consent form and asked to click “agree” if they wanted to participate. Consented individuals were given the opportunity to complete the baseline survey and, subsequently, enroll in the trial by providing information needed to receive intervention materials (e.g., email address). The start of recruitment and enrollment was delayed due to COVID-19 and occurred on a rolling basis. The original plan for a 6-month enrollment period was extended to 9 months (17 May 2023 to 26 February 2024) due to a slower enrollment rate and higher attrition rate than anticipated and to ensure sufficient power for all data collection points.

### 2.2. RCT Design

After the participants completed the online baseline survey and enrollment procedures for the intervention, they were systematically randomized by computer with alternating assignment to the experimental or control group. The participants began receiving intervention materials immediately after randomization. Each week, for 8 weeks, the participants received a new set of electronic intervention materials (i.e., a brief information guide as well as a tracker where they could list guide-related goals and track progress toward them) and ~4 SMS nudges. Additionally, materials for school-age children were available electronically and also mailed to their residences weekly for 7 weeks. The participants were encouraged to spend about 15 min reviewing the intervention materials, share the materials designed for their children with their children, think about changes like those suggested in the materials that could help their families, and implement 1 or 2 easy, quick, low- or no-cost changes in their homes. At week 9, the participants were invited to complete the online post-survey. Approximately 4 to 10 weeks after completing the post-survey, the participants were invited to complete the follow-up survey. Only the findings from the baseline and post-intervention data collection periods are reported in this paper.

The intervention materials for each treatment group provided distinctly different, non-overlapping content. The experimental group received materials focused on one aspect of the weight-related home environment and lifestyle (i.e., overview of parenting school-age kids for good health, fruit/vegetable intake, sugar-sweetened beverage intake, portion size control, family meals, breakfast intake, physical activity level, and sleep hygiene) that parents could re-shape. For details on the experimental intervention development, see Supplementary Material-1 in Byrd-Bredbenner et al. [86]. The attention control group received materials focused on home safety (i.e., overview of home safety, indoor air quality, mold and moisture, hazardous household products, carbon monoxide, foodborne illness, and refrigerator temperatures).

### 2.3. Data Collection Instruments

The study survey, “Home Obesogenicity Measure of EnvironmentS”-2 (HOMES-2), was used to collect data electronically at all data collection points (see Supplementary Material 2 in Byrd-Bredbenner et al. [86]). As indicated in the study protocol [86], the survey collected 4 categories of data: sociodemographic characteristics of the participant, child, partner/spouse (if applicable), and household; participant healthy weight-related behavior (HWRB) cognitions; weight-related behaviors of the participant and child (dietary intake, physical activity, and sleep behaviors); and weight-related home environment characteristics. For items pertaining to children, the participants were instructed to report on one of their children between the ages of 6 and 11 years. In cases where the participants had more than one child between these ages, they were asked to report on the child born closest to a randomly selected time/date (i.e., noon on 7 June).

#### 2.3.1. Sociodemographic Characteristics

Sociodemographic characteristics of the child, participant, and spouse/partner (if applicable) included age, race, and biological sex. The participants also reported their highest level of education, employment status, and marital status. The participants with a spouse/partner also reported their spouse/partner’s highest level of education and employment status. Household characteristics included the number of children under 18 years old in the household, socioeconomic status, food insecurity risk scale [108], and the region of the U.S. where the family resided. A proxy for family socioeconomic status was determined by comparing the median community income in a participant’s home ZIP code to the median state income, with 0 points awarded when the median community income was below the median state income and 1 point awarded when the median community income equaled or exceeded the median state income, with median incomes based on U.S. Census Bureau ACS 5-Year data [109,110].

#### 2.3.2. Healthy Weight-Related Behavior Cognition Scales

HWRB cognitions, as previously described in the study protocol [86], were evaluated using 5-point Likert-type responses (i.e., strongly disagree, disagree, neutral, agree, strongly agree; scored 1 to 5, respectively), with higher scores indicating more positive expression of the cognition. The scales included Outcome Expectations for HWRB, Importance Placed on HWRB for Children, Encouragement and Facilitation of Children’s HWRB, Perceived Effort of Engaging Children in HWRB, Modeling of HWRB, Importance Placed on Modeling HWRB, Self-efficacy for Promoting Children’s HWRB, Self-Efficacy for Personally Engaging in HWRB, Importance Placed on HWRB for Self, and Family Support for HWRB.

#### 2.3.3. Weight-Related Behaviors

The parent and child weight-related behaviors assessed were physical activity level using the Streamlined, Enhanced Self-Report Physical Activity Measure [111]; sleep duration and sleep quality using components from the Pittsburgh Sleep Quality Index [112,113]; and dietary intake behaviors related to breakfast, fruits and vegetables, and sugar-sweetened beverages. Three items documented days/week that breakfast was eaten at all, eaten at home, and eaten from restaurant/convenience store purchases [86]. The 7-item Block Fruit–Vegetable Screener estimated average daily servings of fruits and vegetables [114,115,116]. The 6-item HOMES Survey beverage scale estimated average daily servings of sugar-sweetened beverages [64]. Weight-related parenting behaviors evaluated included days/week that parents involved children in food and mealtime activities and child feeding practices (i.e., pressuring children to not waste food (1 item), pressuring children to eat nutrient-dense food (2 items; Cronbach α = 0.81), restricting access to low-nutrient-density foods (2 items; Cronbach α = 0.83), and instrumental feeding strategies (2 items; Cronbach α = 0.82) [86]. Higher scores for weight-related behaviors reflect greater expression of the behavior.

#### 2.3.4. Home Environment

Weight-related characteristics of the home environment examined were support for weight-protective practices in the home environment, home food availability, and the home physical activity environment. Higher scores reflect home environment characteristics more supportive of healthy weights. Support for weight-protective practices was assessed for adequate sleep, portion control, and family mealtime. Home availability of foods (i.e., breakfast foods, fruits and vegetables, sugar-sweetened beverages, breakfast foods) were assessed using 2 indicator items to determine availability of breakfast foods in the home [86]: the Block Fruit–Vegetable Screener, as adapted for the HOMES survey to capture the total servings of fruits/vegetables available in the home per household member per day [64,117], and sugar-sweetened beverage availability using HOMES survey items, expanded to include prepared smoothies [64]. Fruit/vegetable accessibility within the home and neighborhood environment was assessed using items from the Perceived Nutrition Environment Measures [86]. The home physical activity environment was evaluated with scales from the HOP-UP questionnaire [118], modified slightly to make the items applicable to school-age children (i.e., include play equipment examples that are appropriate for school-age children). In addition, an indicator item that determined the total number of media devices available in children’s bedrooms was included.

### 2.4. Data Analysis

The main study outcomes were family meal frequency as well as child dietary intake, physical activity, and sleep behaviors. The secondary outcome measures were participant HWRB cognitions (precursors to behavior). SPSS software version 30.0 (IBM Corporation, Chicago, IL, USA) was used to conduct statistical analyses. Descriptive statistics were calculated to describe sociodemographic characteristics as well as scores for each outcome measure stratified by treatment group of the enrolled post-survey completers. To determine sociodemographic characteristic differences between the enrolled post-survey completers and non-completers, independent two-tailed *t*-tests for continuous variables and chi-square tests for categorical variables were calculated. Independent two-tailed *t*-tests were also performed to compare sociodemographic characteristic differences by treatment groups. For all outcome measures, paired *t*-tests were conducted to determine within-group mean differences from baseline to post-survey. Cohen’s d calculated effect sizes for continuous variables, while Cramer’s V calculated effect sizes for categorical variables. For Cohen’s d and Cramer’s V, 0.15, 0.3, and 0.45 were set as the benchmarks for small, medium, and large effect sizes, respectively [119,120,121,122]. To determine whether the outcomes were significantly different between groups at post-survey, an Analysis of Covariance (ANCOVA), controlling for baseline scores, was conducted. Effect sizes for ANCOVA were calculated as partial eta-squared values, with 0.01, 0.06, and 0.14 serving as the thresholds for small, medium, and large effect sizes, respectively [123]. All effect sizes are stated in tables. For brevity in reporting, only medium and large effect sizes are stated for significant differences in Section 3 Results below. Due to the numerous comparisons planned, the study protocol stated the Benjamini–Hochberg procedure would be applied by category of data collected and comparison (i.e., baseline vs. post within treatment group, group x time) with a false discovery rate (FDR) of 5% to reduce the risk of type I error [124]. However, the rigid application of this procedure in exploratory studies has been questioned, particularly given concerns about reduced statistical power and increased risk of Type 2 error [125,126]. Some experts further argue that an FDR of 5% may be overly conservative for many experiments and suggest that a threshold between 0.10 and 0.20 may be more appropriate [127]. Additionally, there is limited consensus regarding the routine use of procedures like Benjamini–Hochberg in educational or behavioral interventions [126]. To balance the risks of false positive and false negative findings, the protocol was modified as suggested by McDonald [127]. Raw *p*-values were reported for all significance tests, and instances in which statistical significance was no longer retained after applying Benjamini-Hochberg procedures at FDR thresholds of 0.05 and 0.10 are clearly annotated in the tables and the Section 3 Results.

## 3. Results

**Figure 1:**
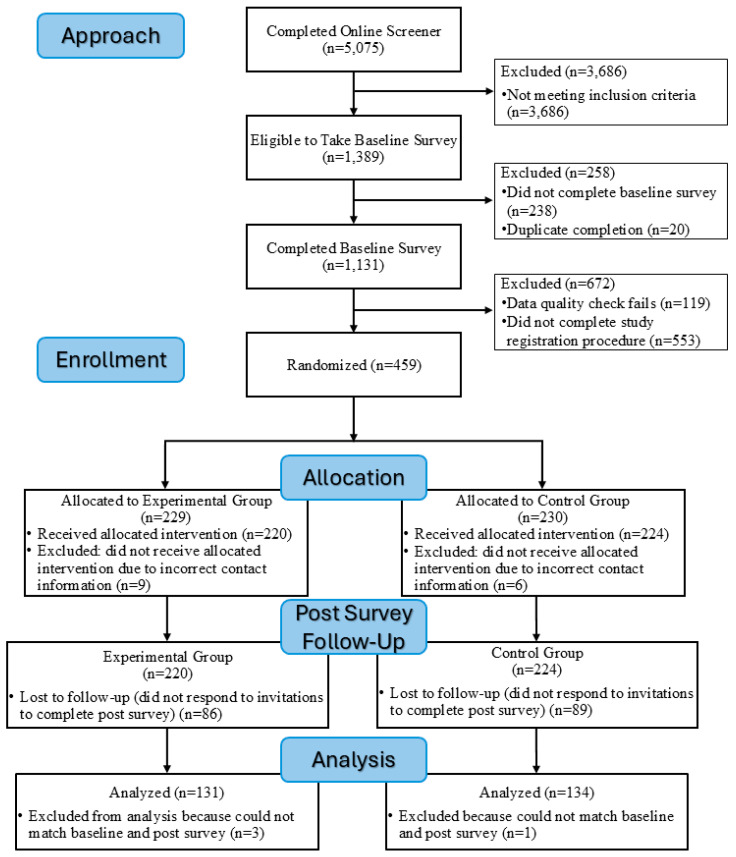
CONSORT flow diagram.

CONSORT guidelines were used to create a participant flow diagram and report RCT enrollment and retention data [128,129]. The Consort Flow Diagram (Figure 1) indicates that a total of 5075 individuals completed the study screener. Of these, 3686 did not meet the study inclusion criteria. Of the 1389 who met study eligibility criteria, 238 were excluded because they did not consent to participate and/or complete the baseline survey, and 20 were excluded because they were duplicate completions. Of the remaining 1131 eligible, consenting individuals, 119 were excluded due to failed survey data quality checks (e.g., inconsistent answers to items measuring the same concept, did not meet the minimal likely survey completion time, all items on a survey page answered the same), and 553 did not complete the study registration procedures and could not be randomized. A total of 459 participants comprised the final randomized sample and were systematically allocated by computer to the experimental group (n = 229) or control group (n = 230). Nine experimental group participants and six control group participants did not receive the allocated intervention due to providing incorrect contact information and thus were excluded. At post-survey follow-up, 86 of the remaining 220 experimental group participants and 89 of the remaining control group participants were lost (i.e., they did not respond to repeated invitations to complete the post-survey). Further, three experimental group participants and one control group participant were excluded because their baseline and post-survey data could not be definitively matched. The final analytic sample was 131 participants in the experimental group and 134 participants in the control group.

### 3.1. Sociodemographic Characteristics

Table 1 compares differences in sociodemographic characteristics of the 265 randomized participants who completed the post-survey with the 175 randomized participants lost to post-survey follow-up. Comparison tests indicate these two groups did not differ significantly in any sociodemographic characteristic, including education level and number of parents in the household, after Benjamini–Hochberg procedures were applied.

A comparison of sociodemographic characteristics by treatment group (Table 2) using independent *t*-tests for continuous variables and chi-square analyses for categorical variables indicates that the groups are comparable. Mean participant age was about 38 years, with most being White, having at least some post-secondary education, being employed, and living in dual-parent households. Spouse/partner data for the two groups were similar, with a mean spouse age of 39 years. Most were White, had at least some post-secondary education, and were employed full-time. Children in the two treatment groups were of similar mean age of approximately 9 years, about equally distributed by sex, and were mostly White. Household characteristics were also similar between the treatment groups. Households averaged about two children under the age of 18 years, lived in ZIP code areas that were below the median income for their state, had a low–moderate food insecurity risk, and lived in regions geographically distributed throughout the United States.

### 3.2. Healthy Weight-Related Behavior Cognitions

The data presented in Table 3 indicate that all HWRB scales had high Cronbach α scores. Comparing within-group differences in HWRB cognitions using one-tailed paired *t*-tests indicates that, between baseline and post-survey, the experimental group improved significantly on all cognitions (with mostly medium effect sizes) except Perceived Effort of Engaging Children in HWRB and Family Support for HWRB. In contrast, only the Modeling of HWRB and Self-Efficacy for Personally Engaging in HWRB scales improved over time in the control group, but neither scale remained significant after applying Benjamini–Hochberg procedures. ANCOVA, controlling for baseline scores, revealed a significant difference between treatment groups at post-survey for the Outcome Expectations for HWRB and Importance Placed on HWRB for Self (medium effect size) cognition scales; only the Importance Placed on HWRB for Self scale remained significant after Benjamini–Hochberg procedures.

### 3.3. Weight-Related Behaviors

As reported in Table 4, between the baseline and post-survey, the experimental group participants significantly improved weight-related behaviors, specifically physical activity level, sleep quality, and fruit/vegetable intake. Additionally, children of the experimental group participants significantly improved fruit/vegetable intake between the baseline and post-survey. The control group participants decreased their intake of sugar-sweetened beverages over time; however, after applying Benjamini–Hochberg procedures, this change was not statistically significant. ANCOVA controlling for baseline scores showed a significant treatment group difference at post-survey for daily fruit/vegetable servings reported for parents and children; statistical significance was sustained after Benjamini–Hochberg procedures at the 10% rate for child fruit/vegetable servings only.

Paired one-tailed *t*-test results for the weight-related parenting behaviors presented in Table 5 show that between baseline and post-survey, the experimental group significantly increased the frequency at which it involved children in family meal planning and preparation. The control group reduced Restricting Children’s Access to Low-Nutrient-Density Foods over time; however, this was no longer statistically significant after Benjamini–Hochberg procedures. ANCOVA controlling for baseline scores indicated no treatment group differences at post-survey.

### 3.4. Home Environment

As shown in Table 6, total family meals per week rose for both treatment groups, but not significantly. For the experimental group, paired one-tailed *t*-test comparisons of baseline and post-survey data revealed Availability of Foods for Breakfast, Availability of Foods for Child to Prepare for Breakfast, Fruit/Vegetable Accessibility at Home, and Fruit/Vegetable Accessibility in Neighborhood all increased, though after applying Benjamini–Hochberg procedures, only Availability of Foods for Child to Prepare for Breakfast remained significant. The significant differences noted in the control group over time (i.e., Support for weight-protective practices in the home: Portion Control and Positive Family Mealtime Setting and Neighborhood Space and Supports) were not sustained after Benjamini–Hochberg procedures. ANCOVA, with baseline scores as the covariate, revealed no treatment group differences at post-survey.

## 4. Discussion

The HomeStyles-Child intervention was designed to help families with children in the middle childhood years gain relevant, practical, non-judgmental obesity prevention knowledge, skills, and confidence needed to re-shape home environments and weight-related lifestyle practices to be more supportive of optimal family health and reduce the risk of obesity [19,57]. HomeStyles-Child engaged parents and middle childhood youth as partners in home-based health promotion and obesity risk reduction efforts. The HomeStyles-Child RCT investigated the effect of participation in this intervention on the weight-related cognitions, lifestyle practices, and home environments of parents and their middle childhood children.

### 4.1. Healthy Weight-Related Behavior Cognitions

Social Cognitive Theory guided the development of the HomeStyles-Child intervention. Care was taken when creating the intervention to incorporate strategies for building parent beliefs that the HWRB promoted in the intervention would lead to positive outcomes. The intervention also consistently reinforced to parents that the importance they place on these behaviors, their effort, encouragement and facilitation of children’s performance of these behaviors, and their modeling of these behaviors was critical to their children actually engaging in these behaviors. The intervention was also designed to build parent self-efficacy so that they, themselves, could perform HWRBs and facilitate their children’s performance [86]. Study findings demonstrated that the HomeStyles-Child intervention did build positive outcome expectations, increased the importance parents placed on HWRBs for children and themselves, boosted parent modeling and value placed on modeling of these behaviors, and increased parent confidence in their ability to engage in HWRB themselves and support their children’s performance. HWRB cognitions were secondary outcome measures for this RCT because cognitions are strong predictors of behavior [132,133].

### 4.2. Weight-Related Behaviors

Lifestyle practices (i.e., behaviors) are the main outcome measures of this RCT, including family meal frequency, dietary intake, physical activity, and sleep [86]. Family mealtime frequency is positively correlated with an array of physical and mental health benefits, such as higher diet quality, more servings of fruits/vegetables, healthier body weight, better emotional well-being, and stronger family bonds [134,135,136,137,138,139,140,141,142,143,144]. Because of these many benefits, the HomeStyles-Child intervention promoted frequent family meals in positive social environments [86]. At baseline, both study groups reported positive mealtime spaces and supports and ate nearly 12 family meals per week. Over time, weekly family mealtime frequency rose by two-thirds of a meal in the experimental group—about the same increase noted in the days/week children were involved in family meal planning and preparation. Frequent family meals and child involvement in family meal planning and preparation are key messages in the HomeStyles-Child intervention materials. The finding that family meal frequency rose in tandem with child involvement in family meal planning and preparation may have contributed to this increase in family meal frequency [145]. Although the increase in family mealtime frequency was not significant, it is important to consider that the typical meal pattern in the U.S. generally includes breakfast, lunch, and dinner, which offers 21 mealtimes per week for the family to dine together. However, parent employment and children’s schooling often limit the possibility of eating the midday meal (lunch) together to just weekend days, thereby reducing possible family meals to 16/week. Work, school, and after-school schedules may further reduce family meal opportunities, which have been observed to decline as children get older [146]. If 16 meals is used as the maximum possible weekly goal, the study groups ate 75% of their meals together as a family at the study outset, leaving little room for improvement. However, the experimental group did increase family meals over time by 4%. The lack of a significant increase in family mealtime frequency is likely due to a ceiling effect; families simply had little or no room for growth.

#### 4.2.1. Dietary Behaviors

HomeStyles-Child is a childhood obesity prevention program; thus, changes in child dietary intake, physical activity, and sleep behaviors were the other main study outcomes. These outcomes are based on key HomeStyles-Child messages related to increasing fruits/vegetables, reducing sugar-sweetened beverage intake, eating breakfast daily, increasing physical activity, and getting adequate and good-quality sleep. Concerning dietary practices, the experimental group parents and their children both increased their fruit/vegetable intakes by nearly 0.5 servings/day, pushing them close to the five-a-day recommendation [147]. These fruit/vegetable intake improvements support health status and healthy body weight [148,149,150,151,152,153,154,155,156,157,158] and likely were facilitated by the home environment, specifically adequate fruit/vegetable availability within the homes of the participants, along with the increased fruit/vegetable accessibility in participant homes and neighborhoods. Indeed, research indicates fruit/vegetable intake is strongly associated with household availability of these foods [159,160,161]. The increased involvement of children in family meal planning and preparation also may have contributed to the increase in daily fruit/vegetable intake [145]. The significant improvements in participant HWRB cognitions also may have helped facilitate increased fruit/vegetable intakes, in that research indicates that parent knowledge of fruit/vegetable benefits (i.e., outcome expectations) [162], as well as their encouragement and modeling of fruit/vegetable intake, promote children’s fruit/vegetable intake [160,163]. Similar to family meal frequency, there was little room for improvement in fruit/vegetable intake at baseline to reach recommended intakes, yet the experimental group participants and children did increase their daily servings.

Sugar-sweetened beverages are a primary source of added sugars in the U.S. diet, providing 66% of children’s total added sugar calories [164,165]. Intake is positively correlated with body weight, along with metabolic syndrome, cardiovascular disease, type 2 diabetes, and bone fracture risk [166,167,168,169,170,171,172,173]. These beverages also displace nutrient-dense beverages, especially dairy milk [174,175]. Hence, reducing the intake of sugar-sweetened beverages is an important behavior to address in obesity prevention programs [176]. The intakes of these beverages were fairly low at baseline (1.22 servings for children and 1.64 servings for adults). A small, non-significant reduction (0.10 serving) was noted in the intake of these beverages over time by the experimental group participants or children. This minor reduction in sugar-sweetened beverage intake paralleled similar reductions noted in the availability of these drinks in the home over time, as well as having little room for reductions (floor effect). Interestingly, the control group participants decreased their intake of sugar-sweetened beverages by about 0.25 servings, which, at post-survey, was equivalent to the unchanged baseline and post-survey intakes of the experimental group. Although the attention control intervention did not address sugar-sweetened beverage intake, the decline in intake of these beverages is consistent with national trends [147].

The participants and children ate breakfast most days of a week at baseline and post-survey. A slight, non-significant upward trend in the days/week breakfast was eaten at all and eaten at home was noted in the experimental group participants. This improvement aligns with the increase in the home environment availability of foods for breakfast and the availability of foods for children to prepare for breakfast. Further emphasis on parental modeling of eating breakfast to children is important to support because regular intake of this meal is associated with healthy body weight, healthier diets overall, and better academic performance [177,178,179,180,181,182,183,184,185,186,187,188]. Plus, building strong breakfast eating habits before adolescence helps protect against the precipitous decline in breakfast intake commonly observed during the teenage years [189,190].

#### 4.2.2. Physical Activity Behaviors

Physical activity level is inversely related to waist circumference and body weight [191,192]. About four out of 10 children in the U.S. meet the recommendations for 1 h of physical activity daily [193]. For adults, the proportion drops to one in four who meet physical activity guidelines for aerobic and muscle-strengthening activities [194,195]. Parental beliefs about active playtime, coupled with their guidance, encouragement, and support of children, have a strong influence on the active play behaviors of their school-aged children [196,197,198,199,200,201]. The participants reported that good physical activity spaces and supports were available inside their homes and in outdoor/yard areas, with neighborhood space and supports available to a moderate extent. HomeStyles-Child provided strategies for increasing space and supports for physical activity in and around the home, as well as ideas for incorporating neighborhood facilities (e.g., parks) into family physical activity routines. The small increase in space inside the home and in the neighborhood over time may have occurred because the participants learned strategies for making space and supports available inside the home for physical activity and greater awareness of supports in the neighborhood. The study findings suggest the HomeStyles-Child intervention promoted improvements in physical activity over time in the participants. This increase in physical activity is an important outcome; however, the participant’s physical activity level only reached the mid-point of the scale at post-survey, thereby indicating a need to encourage even greater physical activity among parents. This need is particularly critical given the positive correlation between parent behavioral modeling of physical activity and child physical activity levels, the non-significant increase in activity level of children in the experimental group, and the tendency for physical activity to decline as children get older [22,23,83,85,202,203,204,205,206,207]. The HomeStyles-Child intervention promoted increased physical activity using fun, low- to no-cost, easily implemented and scheduled family activities along with reduced leisure-time use of electronic screen devices (e.g., phones, television, computers)—strategies fully responsive to cognitions, barriers, and supports for active play behaviors recommended by parents and school-age children [208]. A slight decline in the number of media devices in the bedrooms of the participants’ children is encouraging and was promoted in the HomeStyles-Child intervention. However, the lack of significant physical activity increases may indicate a need for greater emphasis on this behavior change and/or consideration of how other, unrelated factors may impede implementing these behaviors.

#### 4.2.3. Sleep Behaviors

Adequate sleep is needed for normal growth and development during childhood and offers physical and mental health benefits at all life stages [209,210,211,212,213,214,215]. The sleep duration remained unchanged in the participants, perhaps because it was within the sleep recommendation guidelines of 7 to 9 h/night for adults at baseline. However, similar to other research findings [216], sleep duration was less than the recommended 9 to 12 h/night for children aged 6 to 12 years [217]. Sleeping less than the recommended amounts increases children’s risk for impaired cardiometabolic function, hypertension, and overweight [218,219,220]. A positive finding is that the participants reported having a set bedtime in their households more than 5 days per week. Another positive is the increase in sleep quality among the participants. This improvement suggests the healthier bedtime practices and sleep hygiene promoted in the HomeStyles-Child intervention were implemented, which may, over time, lead to longer sleep duration in parents as well as their children who observe and model parental sleep-related practices [83,132,221]. Also, Social Cognitive Theory posits that the positive improvements noted in participant HWRB cognitions, such as outcome expectations, may have supported the sleep quality improvements [83,84,133].

#### 4.2.4. Parenting Behaviors

Weight-related parenting behaviors, in addition to involving children in food and mealtime activities, included the participants’ child feeding practices. HomeStyles-Child is one of the few interventions for children beyond the preschool years that have investigated child feeding practices, such as those related to pressuring children to eat particular foods (e.g., fruits/vegetables), rewarding children for eating foods like fruits/vegetables, or restricting access to highly palatable foods [79]. Although HomeStyles-Child promoted positive feeding strategies, these parenting behaviors did not change over time. Although it is unclear why there was no change, it could be that these particular child feeding practices become less important as children get older and more independent. Also, the economic challenges of the COVID-19 pandemic may have prompted parents to continue to pressure children not to waste food. Future research investigating parent feeding practices in middle childhood could help identify parent concerns for this age group, along with age-appropriate issues to address in interventions.

### 4.3. Strengths and Limitations

Every RCT has strengths and limitations. Care was taken to minimize limitations and, whenever possible, re-configure them to be strengths. Although the use of self-report survey instruments may be a limitation, many aspects of the survey could only be collected by self-report, such as cognitions, which are internally held beliefs. Additionally, all components of the survey were carefully designed and tested to ensure they collected data that were both valid and reliable, in a user-friendly manner using strategies that minimized participant burden and promoted survey completion [86]. The survey instruments were also well matched to the online intervention delivery mode. In fact, an online data collection mode allows participants to respond privately, remotely, and with relative anonymity, which helps minimize social desirability risk [222]. Social desirability risk was further reduced by stating in the survey preamble that all responses are acceptable, normal, and confidential [222,223]. Other advantages of the data collection mode and intervention delivery were that they permitted nationwide recruitment of participants.

The intervention was designed to be responsive to quality-of-life factors identified by parents and school-age children and convenient for participants to access. Further strengths of this protocol were the use of a bona fide, credible, structurally equivalent attention control group treatment comparable in time commitment and non-specific effects, which, along with recruitment materials and distractor items on the survey, served to blind participants to treatment assignment.

Attrition in RCTs, especially with time-stressed audiences like parents and, in this case, coping with COVID-19 effects, presented challenges to ensuring adequate sample size. To help contain the attrition rate, participants received a stipend that rose in value at each data collection point to compensate for their time spent completing the survey. In addition, the recruitment period was extended in an attempt to ensure an adequate sample size. The study sample size reached 99% of the *a priori* target calculated to detect statistical significance at *p* < 0.05 with 80% power. However, the study was inadequately powered for the application of Benjamini–Hochberg procedures and may have precipitated Type 2 errors. To address this limitation, *p*-values for the tests of significance and effects of Benjamini–Hochberg procedures at the 5% and 10% false discovery rates were reported.

## 5. Conclusions

HomeStyles-Child is one of the few interventions for families with middle childhood youth [19,57,58,59]. It has the potential to help ameliorate obesity in middle childhood youth and, by extension, other family members. This RCT has demonstrated the feasibility of online delivery of a health promotion intervention that may inform the development of interventions targeting other age groups and health outcomes. The results indicate that the HomeStyles-Child intervention was effective in improving parent HWRB cognitions, which are predictors of behavior change [83,132,133]. Additionally, improvements in parent and child health-related behaviors were observed. These improvements occurred during a time when families faced unprecedented and extraordinary economic and social stresses associated with the COVID-19 pandemic, leading one to wonder what the outcome would have been in less challenging circumstances.

## Figures and Tables

**Table 1:** Baseline sociodemographic characteristics comparison of randomized participants completing the post-survey and those lost to follow-up.

Characteristic	Post-Survey Completers	Lost to Follow-Up	χ^2^ or *t*-Value *	*P*	Cohen’s *d* or Cramer’s *V* #
	Mean ± SD or n (%)	Mean ± SD or n (%)			
**Participant**	**(n = 265)**	**(n = 175)**			
Age	37.99 ± 5.71	37.65 ± 5.96	0.607 **	0.544	0.060
Race (White) ^A^	185 (70)	110 (63)	2.307	0.129	0.072
Education Level ^B^ High school or less Some post-secondary education College graduate or higher	67 (25) 92 (35) 106 (40)	47 (27) 81 (46) 47 (27)	8.924	0.012 †	0.142
Employment Status ^C^ Does not work Part-time Full-time	96 (36) 71 (37) 98 (37)	67 (38) 53 (30) 55 (31)	1.512	0.470	0.059
Dual-Parent Household ^D^	207 (78)	120 (69)	5.028	0.025 †	0.107
**Spouse**	**(n = 207)**	**(n = 120)**			
Age	39.37 ± 9.98	39.32 ± 8.65	0.053 **	0.958	0.006
Sex (Male)	182 (88)	106 (88)	0.012	0.912	0.006
Race (White) ^A^	75 (38)	45 (36)	0.053	0.819	0.013
Education Level ^B^ High school or less Some post-secondary Education College graduate or higher	80 (39) 62 (30) 65 (31)	50 (42) 37 (31) 32 (27)	0.764	0.682	0.048
Employment Status ^C^ Does not work Part-time Full-time	18 (9) 32 (15) 157 (76)	8 (7) 27 (23) 85 (71)	0.423	0.673	0.048
**Child** ††	**(n = 257)**	**(n = 163)**			
Age	9.11 ± 1.71	9.02 ± 1.59	0.536 **	0.592	0.054
Sex (Male) ^E^	144 (56)	94 (58)	0.109	0.741	0.016
Race (White) ^A^	160 (62)	88 (54)	2.820	0.093	0.082
**Household**					
# Children Under 18 Years in Household	2.28 ± 1.09	2.36 ± 1.01	0.833 **	0.405	0.080
Socioeconomic Status [109,110] ^F^	0.42 ± 0.49	0.33 ± 0.47	1.866 **	0.063	0.180
Food Insecurity Risk [108] ^G^	2.10 ± 1.01	2.24 ± 1.06	1.314 **	0.190	0.129
Region of Residence [130] ^H^**			0.247	0.970	0.024
Northeastern	42 (16)	28 (16)			
Midwestern	71 (27)	48 (27)			
Southern	119 (45)	75 (43)			
Western	33 (12)	24 (14)			

* Independent two-tailed *t*-tests were conducted for all continuous variables and marked with **. Chi-square tests were used for categorical variables. # Effect size was calculated using Cohen’s d for continuous variables. Cramér’s V was used for categorical variables. † Benjamini–Hochberg procedures with a 5% or 10% false discovery rate indicate *P* is not significant. †† A child was excluded from analysis when data quality checks indicated the child was not in the age range targeted (i.e., ages 6 to 11 years) and/or failed to verify that the same child was being reported on in the baseline and post-surveys (e.g., child name, age, sex, race/ethnicity were inconsistent). ^A^ Race/ethnicity was coded as: 0 = White and 1 = Non-White (Black or African American; American Indian or Alaskan Native; Asian Indian; Asian; Pacific Islander, and Other specified by the participant). ^B^ Education level was coded as: 1 = high school or less, 2 = some college, and 3 = college graduate. ^C^ Employment status was coded as 1= does not work for pay, 2 = part-time (<30 h of paid employment/week), and 3 = full-time (≥30 h of paid employment/week). ^D^ 1 = Single-parent (single, divorced, widowed), 2 = dual-parent (married, living with partner). ^E^ Sex was coded as: 0 = Boy, 1 = Girl. ^F^ Socioeconomic status (determined using participants’ home ZIP codes to assess the median community income) coded as 0 = zip code median income < median income for the state and 1 = zip code median community income ≥ median for the state, based on U.S. Census Bureau ACS 5-Year data [109,110]. ^G^ Two-item scale with 4-point Agreement Rating, namely, definitely false, mostly false, mostly true, and definitely true; scored 1 to 4, respectively. Items averaged with higher scores indicating greater food insecurity risk [108]. ^H^ Region of residence based on predetermined grouping as defined by the U.S. Census Bureau [130] Northeastern: Connecticut, Maine, Massachusetts, New Hampshire, New Jersey, New York, Pennsylvania, Rhode Island, Vermont. Midwestern: Illinois, Indiana, Iowa, Kansas, Michigan, Minnesota, Missouri, Nebraska, North Dakota, Ohio, South Dakota, and Wisconsin. Southern: Alabama, Arkansas, Delaware, District of Columbia, Florida, Georgia, Kentucky, Louisiana, Maryland, Mississippi, North Carolina, Oklahoma, South Carolina, Tennessee, Texas, Virginia, West Virginia. Western: Alaska, Arizona, California, Colorado, Hawaii, Idaho, Montana, Nevada, New Mexico, Oregon, Utah, Washington, and Wyoming.

**Table 2:** Baseline sociodemographic characteristics of randomized participants completing the post-survey by treatment group.

Characteristic	Experimental	Control	χ^2^ or *t*-Value *	*P*	Cohen’s *d* or Cramer’s *V* #
	Mean ± SD or n (%)	Mean ± SD or n (%)			
**Participant**	**(n = 131)**	**(n = 134)**			
Age	38.27 ± 5.55	37.72 ± 5.86	0.796 **	0.427	0.098
Race (White) ^A^	86 (66)	99 (74)	2.130	0.144	0.090
Education Level ^B^ High school or less Some post-secondary education College graduate or higher	29 (22) 46 (35) 56 (43)	38 (28) 46 (34) 50 (37)	1.515	0.469	0.076
Employment Status ^C^ Does not work Part-time Full-time	43 (33) 36 (28) 52 (40)	53 (40) 35 (26) 46 (34)	1.389	0.499	0.072
Dual-Parent Household ^D^	98 (75)	109 (81)	1.654	0.198	0.079
**Spouse**	**(n = 98)**	**(n = 109)**			
Age	39.39 ± 9.97	39.36 ± 10.03	0.022 **	0.983	0.003
Sex (Male) ^E^	84 (86)	98 (90)	0.855	0.355	0.064
Race (White) ^A^	60 (61)	72 (66)	0.521	0.470	0.050
Education Level ^B^ High school or less Some post-secondary education College graduate or higher	31 (32) 35 (36) 32 (33)	49 (45) 27 (25) 33 (30)	4.526	0.104	0.148
Employment Status ^C^ Does not work Part-time Full-time	11 (11) 13 (13) 74 (76)	7 (6) 19 (17) 83 (76)	1.951	0.377	0.097
**Child** †	**(n = 126)**	**(n = 131)**			
Age	9.10 ± 1.71	9.13 ± 1.71	0.163 **	0.871	0.020
Sex (Male) ^E^	67 (53)	77 (59)	0.819	0.366	0.056
Race (White) ^A^	70 (56)	90 (69)	4.724	0.030 ††	0.136
**Household**	**(n = 131)**	**(n = 134)**			
# Children Under 18 Years in Household	2.24 ± 1.12	2.31 ± 1.07	0.571	0.568	0.070
Socioeconomic Status Proxy [109,110] ^F^	0.47 ± 0.50 (53% below median)	0.37 ± 0.49 (63% below median)	1.527 **	0.128	0.188
Food Insecurity Risk [108] ^G^	2.08 ± 0.96	2.13 ± 1.06	0.437 **	0.663	0.054
Region of Residence [130] ^H^			1.585	0.663	0.077
Northeastern	22 (17%)	20 (15%)			
Midwestern	32 (24%)	39 (29%)			
Southern	58 (44%)	61 (46%)			
Western	19 (15%)	14 (10%)			

* Independent two-tailed *t*-tests were conducted for all continuous variables and marked with **. Chi-square tests were used for categorical variables. # Effect size was calculated using Cohen’s d for continuous variables. Cramér’s V was used for categorical variables. † Children were excluded from analysis when the age reported was outside the study range (i.e., <6 and >12 years of age). †† Benjamini–Hochberg procedures with a 5% or 10% false discovery rate indicate *P* is not significant. ^A^ Race/ethnicity was coded as: 0 = White and 1 = Non-White (Black or African American; American Indian or Alaskan Native; Asian Indian; Asian; Other specified by the participant). ^B^ Education level was coded as: 1 = high school or less, 2 = some post-secondary education, and 3 = college graduate or higher. ^C^ Employment status coded as 1= does not work for pay, 2 = part-time (<30 h of paid employment/week), and 3= full-time (≥30 h of paid employment/week). ^D^ 1 = Single-parent (single, divorced, widowed), 2 = dual-parent (married, living with partner). ^E^ Sex was coded dichotomously as: 0 = Male and 1 = Female. ^F^ Socioeconomic status (determined using participants’ home ZIP codes to assess the median community income) coded dichotomously as 0 = zip code median income < median income for the state and 1 = zip code median community income > median for the state, based on U.S. Census Bureau ACS 5-Year data [109,110]. ^G^ Two-item scale with 4-point Agreement Rating, namely, definitely false, mostly false, mostly true, and definitely true, scored 1 to 4, respectively. Items averaged with higher scores indicating greater food insecurity risk [108]. ^H^ Region of residence based on predetermined grouping as defined by the U.S. Census Bureau [130]. Northeastern: Connecticut, Maine, Massachusetts, New Hampshire, New Jersey, New York, Pennsylvania, Rhode Island, Vermont. Midwestern: Illinois, Indiana, Iowa, Kansas, Michigan, Minnesota, Missouri, Nebraska, North Dakota, Ohio, South Dakota, and Wisconsin. Southern: Alabama, Arkansas, Delaware, District of Columbia, Florida, Georgia, Kentucky, Louisiana, Maryland, Mississippi, North Carolina, Oklahoma, South Carolina, Tennessee, Texas, Virginia, West Virginia. Western: Alaska, Arizona, California, Colorado, Hawaii, Idaho, Montana, Nevada, New Mexico, Oregon, Utah, Washington, and Wyoming.

**Table 3:** Healthy weight-related behavior (HWRB) cognitions by treatment group over time (baseline and post-intervention).

Cognition [86] ^A^	Experimental (n = 131)		Control (n = 134)		Experimental Over Time *		Control Over Time *		ANCOVA ## Group × Time	
	Baseline Mean ± SD (95% CI)	Post Mean ± SD (95% CI)	Baseline Mean ± SD (95% CI)	Post Mean ± SD (95% CI)	*t*-Test (*P*) *df* = 130	*d #*	*t*-Test (*P*) *df* = 133	*d #*	F-Test (*P*) *df* = 1, 263	*ηp^2^*
Outcome Expectations for HWRB (23 items; Cronbach α = 0.93)	4.07 ± 0.52 (3.98–4.16)	4.23 ± 0.56 (4.13–4.33)	4.15 ± 0.57 (4.06–4.25)	4.18 ± 0.56 (4.09–4.28)	3.919 (<0.0001)	0.300	0.709 (0.240)	0.047	4.352 (0.038) †	0.016
Importance Placed on HWRB for Children (13 items; Cronbach α = 0.80)	4.06 ± 0.51 (3.97–4.15)	4.18 ± 0.51 (4.09–4.27)	4.12 ± 0.45 (4.04–4.20)	4.16 ± 0.51 (4.08–4.25)	3.345 (0.0005)	0.239	1.281 (0.101)	0.093	1.558 (0.213)	0.006
Encouragement and Facilitation of Children’s HWRB (12 items; Cronbach α = 0.81)	3.86 ± 0.53 (3.77–3.95)	4.03 ± 0.60 (3.93–4.13)	3.94 ± 0.57 (3.84–4.04)	3.99 ± 0.56 (3.89–4.08)	3.890 (<0.0001)	0.302	0.947 (0.173)	0.088	1.881 (0.171)	0.007
Perceived Effort of Engaging Children in HWRB (12 items; Cronbach α = 0.96)	4.12 ± 0.85 (3.97–4.27)	4.08 ± 1.11 (3.89–4.28)	4.23 ± 0.92 (4.08–4.39)	4.09 ± 0.97 (3.93–4.26)	0.391 (0.652)	0.035	2.227 (0.986)	0.149	0.486 (0.486)	0.002
Modeling of HWRB (9 items; Cronbach α = 0.76)	3.49 ± 0.62 (3.38–3.60)	3.69 ± 0.57 (3.59–3.78)	3.52 ± 0.63 (3.42–3.63)	3.63 ± 0.59 (3.53–3.73)	4.016 (<0.0001)	0.330	1.964 (0.026) †	0.168	1.563 (0.212)	0.006
Importance Placed on Modeling HWRB (eight items; Cronbach α = 0.83)	3.89 ± 0.69 (3.77–4.00)	4.02 ± 0.68 (3.91–4.14)	3.97 ± 0.69 (3.85–4.08)	3.95 ± 0.68 (3.83–4.07)	2.531 (0.006)	0.201	0.330 (0.629)	0.025	3.423 (0.065)	0.013
Self-Efficacy for Promoting HWRB in Children (13 items; Cronbach α = 0.91)	4.04 ± 0.63 (3.93–4.15)	4.19 ± 0.63 (4.08–4.29)	4.17 ± 0.58 (4.07–4.27)	4.15 ± 0.61 (4.05–4.26)	3.265 (0.0007)	0.233	0.387 (0.650)	0.031	3.768 (0.053)	0.014
Self-Efficacy for Personally Engaging in HWRB (10 items; Cronbach α = 0.85)	3.80 ± 0.68 (3.68–3.92)	4.06 ± 0.65 (3.95–4.17)	3.91 ± 0.66 (3.80–4.03)	4.00 ± 0.65 (3.89–4.11)	5.244 (<0.0001)	0.391	1.694 (0.046) †	0.136	3.675 (0.056)	0.014
Importance Placed on HWRB for Self (nine items; Cronbach α = 0.78)	3.74 ± 0.63 (3.63–3.85)	4.00 ± 0.61 (3.90–4.11)	3.76 ± 0.67 (3.65–3.88)	3.82 ± 0.63 (3.71–3.92)	5.417 (<0.0001)	0.419	1.122 (0.132)	0.082	11.182 (<0.001)	0.041
Family Support for HWRB (11 items; Cronbach α = 0.92)	2.44 ± 0.92 (2.28–2.60)	2.44 ± 1.10 (2.25–2.63)	2.26 ± 1.01 (2.09–2.43)	2.38 ± 1.07 (2.19–2.56)	0.064 (0.475)	0.005	1.578 (0.059)	0.112	0.233 (0.630)	0.001

* Paired one-tailed *t*-tests in the expected direction (i.e., upper tail for all scores); effect size calculated with Cohen’s *d*. † Benjamini–Hochberg procedures with a 5% or 10% false discovery rate indicate *P* is not significant. # Effect size was calculated using Cohen’s d for continuous variables. ## ANCOVA: Analysis of Covariance with baseline value as the covariate; effect size calculated with partial eta-squared (*ηp*^2^). ^A^ Five-point Agreement Rating, namely, strongly disagree, disagree, neutral, agree, and strongly agree, scored 1 to 5, respectively. Scale items averaged to create a scale score; higher scores indicate greater expression of the cognition.

**Table 4:** Weight-related behaviors by treatment group and over time (baseline and post-intervention).

Behavior	Experimental		Control		Experimental Over Time *		Control Over Time		ANCOVA ## Group × Time	
	Baseline Mean ± SD (95% CI)	Post Mean ± SD (95% CI)	Baseline Mean ± SD (95% CI)	Post Mean ± SD (95% CI)	*t*-Test (*P*) *df* = 130	*d #*	*t*-Test (*P*) *df* = 133	*d #*	F-Test (*P*) *df* = 1, 263	*ηp* ^2^
**Participant**	**(n = 131)**		**(n = 134)**							
Fruit/Vegetable Servings/Day [114,115,116]	4.33 ± 1.63 (4.04–4.61)	4.72 ± 1.98 (4.38–5.06)	4.15 ± 1.83 (3.83–4.46)	4.16 ± 1.84 (3.85–4.48)	2.612 (0.005)	0.216	0.124 (0.451)	0.008	5.564 (0.019) ††	0.021
Sugar-Sweetened Beverage Servings/Day [64]	1.64 ± 1.62 (1.36–1.92)	1.65 ± 2.22 (1.27–2.04)	1.88 ± 2.17 (1.51–2.25)	1.63 ± 1.98 (1.29–1.97)	0.046 (0.518)	0.004	2.067 (0.020) ††	0.122	−0.978 (0.324)	0.004
Breakfast Consumed (days/week) [86]	5.21 ± 2.28 (4.82–5.61)	5.36 ± 2.05 (5.00–5.71)	4.96 ± 2.34 (4.56–5.35)	4.94 ± 2.34 (4.54–5.34)	0.888 (0.188)	0.067	0.084 (0.533)	0.006	1.559 (0.213)	0.006
Eat Breakfast at Home	4.82 ± 2.24 (4.43–5.20)	4.93 ± 2.06 (4.57–5.29)	4.43 ± 2.29 (4.04–4.82)	4.50 ± 2.32 (4.10–4.90)	0.660 (0.255)	0.053	0.345 (0.365)	0.029	1.003 (0.317)	0.004
Physical Activity Level [111] ^A^	20.40 ± 12.04 (18.32–22.49)	22.73 ± 11.15 (20.81–24.66)	19.06 ± 11.96 (17.02–21.10)	20.07 ± 12.49 (17.94–22.21)	2.337 (0.0105)	0.201	1.233 (0.110)	0.083	2.553 (0.111)	0.010
Sleep Duration (hours/day) [112,113]	7.24 ± 1.69 (6.95–7.53)	7.27 ± 1.20 (7.06–7.48)	7.15 ± 1.04 (6.97–7.33)	7.12 ± 1.28 (6.90–7.33)	0.208 (0.418)	0.021	0.382 (0.648)	0.032	0.781 (0.378)	0.003
Sleep Quality [112,113] ^B^	3.30 ± 0.93 (3.14–3.46)	3.52 ± 0.97 (3.35–3.69)	3.25 ± 0.75 (3.13–3.38)	3.32 ± 0.84 (3.18–3.46)	3.345 (0.0005)	0.233	0.954 (0.171)	0.084	3.605 (0.059)	0.014
**Child** †	**(n = 110)**			**(n = 114)**						
Sleep Quality [112,113] ^B^	4.17 ± 0.74 (4.03–4.31)	4.23 ± 0.71 (4.09–4.36)	4.18 ± 0.66 (4.05–4.30)	4.29 ± 0.71 (4.16–4.42)	0.831 (0.204)	0.075	1.575 (0.059)	0.167	0.517 (0.473)	0.002
Fruit/Vegetable Servings/Day [114,115,116]	4.45 ± 1.79 (4.11–4.70)	4.85 ± 1.71 (4.53–5.18)	4.24 ± 1.96 (3.88–4.6)	4.20 ± 1.86 (3.86–4.55)	2.842 (0.003)	0.230	0.256 (0.601)	0.020	8.176 (0.005)	0.036
Sugar-Sweetened Beverage Servings/Day [64]	1.22 ± 1.19 (1.00–1.45)	1.11 ± 1.43 (0.84–1.38)	1.23 ± 1.21 (1.00–1.45)	1.26 ± 1.58 (0.96–1.55)	1.110 (0.135)	0.084	0.254 (0.600)	0.020	0.879 (0.350)	0.004
Breakfast Consumed (days/week) [86]	6.01 + 1.77 (5.68–6.35)	5.90 + 1.88 (5.44–6.26)	5.97 + 1.69 (5.66–6.29)	5.96 + 1.62 (5.66–6.27)	0.715 (0.762)	0.068	0.058 (0.523)	0.005	0.201 (0.654)	0.001
Physical Activity Level [111] ^A^	25.05 ± 12.19 (22.77–27.35)	26.43 ± 12.51 (24.06–28.79)	23.98 ± 12.73 (21.62–26.34)	23.51 ± 12.09 (21.27–25.75)	1.108 (0.135)	0.112	0.478 (0.683)	0.038	2.874 (0.091)	0.013
Sleep Duration (hours/day) [112,113]	8.69 ± 1.23 (8.46–8.92)	8.79 ± 1.10 (8.58–9.00)	8.83 ± 1.03 (8.64–9.02)	8.75 ± 1.06 (8.56–8.95)	0.845 (0.200)	0.086	0.837 (0.798)	0.071	0.677 (0.411)	0.003

* Paired one-tailed *t*-tests in the expected direction (i.e., upper tail for all scores in the table, except the lower tail was used for eat breakfast at restaurant/convenience store and sugar-sweetened beverage servings/day); effect size calculated with Cohen’s *d*. # Effect size was calculated using Cohen’s d for continuous variables. ## ANCOVA: Analysis of Covariance with baseline value as the covariate; effect size calculated with partial eta-squared (*ηp*^2^). † A child was excluded from analysis when data quality checks failed to verify the same child was being reported on in the baseline and post-surveys (e.g., child name, age, sex, race/ethnicity were inconsistent). †† Benjamini–Hochberg procedures with a 5% or 10% false discovery rate indicate *P* is not significant. ⁋ Benjamini–Hochberg procedures with a 5% false discovery rate indicate P is not significant. Significance remains at a 10% false discovery rate. ^A^ Scores are weighted by exercise intensity, exercise level = (days/week vigorous activities × 3) + (days/week moderate activities × 2) + (days/week walking × 1) + (days/week resistance training × 1) = physical activity score. ^B^ Five-point Rating Scale, namely, very bad, bad, ok, good, and very good, scored 1 to 5, respectively. Higher score indicates better sleep quality.

**Table 5:** Parenting behaviors by treatment group and over time (baseline and post-intervention).

Behavior	Experimental (n = 131)		Control (n = 134)		Experimental over Time *		Control over Time *		ANCOVA ## Group × Time	
	Baseline Mean ± SD (95% CI)	Post Mean ± SD (95% CI)	Baseline Mean ± SD (95% CI)	Post Mean ± SD (95% CI)	*t*-Test (*P*) *df* = 130	*d #*	*t*-Test (*P*) *df* = 133	*d #*	F-Test (*P*) *df* = 1, 263	*ηp* ^2^
Child Involvement in Family Meal Planning and Preparation Behaviors (days/week)	2.65 ± 1.58 (2.37–2.92)	3.02 ± 1.58 (2.74–3.29)	2.82 ± 1.65 (2.53–3.10)	3.00 ± 1.75 (2.70–3.30)	2.561 (0.0058)	0.233	1.386 (0.084)	0.110	0.355 (0.552)	0.001
Child Feeding Practices [64,131] ^A^										
Pressures Children to Not Waste Food (one item, Cronbach α = n/a)	3.91 ± 0.96 (3.74–4.08)	4.01 ± 0.89 (3.85–4.16)	4.05 ± 0.98 (3.88–4.22)	4.13 ± 0.94 (3.97–4.29)	1.155 (0.875)	0.107	0.889 (0.812)	0.085	0.478 (0.490)	0.002
Pressures Children to Eat Nutrient-Dense Food (two items; Cronbach α = 0.81)	2.62 ± 1.18 (2.42–2.83)	2.46 ± 1.15 (2.26–2.66)	2.45 ± 1.16 (2.25–2.66)	2.45 ± 1.22 (2.24–2.66)	1.645 (0.051)	0.138	0.047 (0.519)	0.003	0.763 (0.383)	0.003
Restricts Children’s Access to Low-Nutrient-Density Foods (two items; Cronbach α = 0.83)	3.08 ± 1.04 (2.90–3.26)	2.99 ± 1.10 (2.80–3.18)	3.09 ± 1.07 (2.91–3.28)	2.92 ± 1.07 (2.74–3.10)	0.908 (0.183)	0.086	1.994 (0.024) †	0.160	0.403 (0.526)	0.002
Use Instrumental Feeding Strategies (two items; Cronbach α = 0.82)	2.59 ± 1.09 (2.40–2.78)	2.58 ± 1.19 (2.37–2.78)	2.46 ± 1.10 (2.27–2.65)	2.51 ± 1.19 (2.31–2.72)	0.126 (0.450)	0.010	0.663 (0.746)	0.049	0.045 (0.833)	<0.001

* Paired one-tailed *t*-tests in the expected direction (i.e., lower tail for all scores in the table, except for Child Involvement in Family Meal Planning and Preparation Behaviors); effect size calculated with Cohen’s *d*. # Effect size was calculated using Cohen’s d for continuous variables. ## ANCOVA: Analysis of Covariance with baseline value as the covariate; effect size calculated with partial eta-squared (*ηp*^2^). ^A^ Five-point Agreement Rating, namely, strongly disagree, disagree, neutral, agree, and strongly agree, scored 1 to 5, respectively. Scale item scores averaged to create scale score; higher scores indicate greater expression of the cognition. † Benjamini–Hochberg procedures with a 5% or 10% false discovery rate indicate *P* is not significant.

**Table 6:** Home environment by treatment group over time.

Characteristic	Experimental (n = 131)		Control (n = 134)		Experimental over Time *		Control over Time *		ANCOVA ## Group × Time	
	Baseline Mean ± SD (95% CI)	Post Mean ± SD (95% CI)	Baseline Mean ± SD (95% CI)	Post Mean ± SD (95% CI)	*t*-Test (*P*) *df* = 130	*d #*	*t*-Test (*P*) *df* = 133	*d #*	F-Test (*P*) *df* = 1, 263	*ηp* ^2^
Family Meals per Week	11.79 ± 5.10 (10.91–12.68)	12.46 ± 5.37 (11.53–13.39)	11.65 ± 5.00 (10.79–12.50)	11.81 ± 4.85 (10.99–12.64)	1.513 (0.066)	0.127	0.424 (0.336)	0.033	1.168 (0.281)	0.004
Support for Weight-Protective Practices in the Home [86] ^A^										
Adequate Sleep (set bedtime)	5.19 ± 2.16 (4.82–5.56)	5.25 ± 2.06 (4.90–5.61)	5.28 ± 2.08 (4.92–5.63)	5.25 ± 2.27 (4.87–5.64)	0.373 (0.355)	0.029	0.098 (0.539)	0.010	0.022 (0.882)	<0.001
Portion Control	4.09 ± 0.97 (3.92–4.26)	4.19 ± 0.98 (4.02–4.36)	4.04 ± 1.09 (3.86–4.23)	4.30 ± 0.86 (4.15–4.45)	1.051 (0.148)	0.102	2.387 (0.009) ††	0.259	1.238 (0.267)	0.005
Positive Family Mealtime Setting	4.06 ± 0.84 (3.92–4.21)	4.16 ± 0.74 (4.03–4.29)	4.13 ± 0.85 (3.98–4.27)	4.26 ± 0.71 (4.14–4.38)	1.399 (0.082)	0.125	1.683 (0.047) ††	0.171	0.907 (0.342)	0.003
Space for Family Meals at Home	4.44 ± 0.71 (4.31–4.56)	4.45 ± 0.70 (4.33–4.57)	4.58 ± 0.67 (4.47–4.70)	4.56 ± 0.66 (4.45–4.67)	0.306 (0.380)	0.022	0.413 (0.660)	0.034	0.093 (0.761)	<0.001
Home Food Availability [64,86,117]										
Fruits/Vegetables (servings/household member/day) [64,114,117]	5.84 ± 2.56 (5.40–6.28)	5.39 ± 2.18 (5.01–5.76)	5.60 ± 2.46 (5.18–6.02)	5.19 ± 2.23 (4.81–5.57)	2.430 (0.992)	0.191	2.105 (0.981)	0.175	0.116 (0.734)	<0.001
Sugar-Sweetened Beverage Servings/Household Member/Day [64]	0.62 ± 0.71 (0.50–0.75)	0.55 ± 0.65 (0.44–0.66)	0.59 ± 0.66 (0.48–0.70)	0.67 ± 0.74 (0.54–0.80)	1.040 (0.150)	0.108	1.179 (0.880)	0.113	2.604 (0.108)	0.010
Breakfast [86] ^A^										
Availability of Foods for Breakfast	4.39 ± 0.83 (4.25–4.53)	4.53 ± 0.67 (4.42–4.65)	4.63 ± 0.72 (4.51–4.76)	4.54 ± 0.64 (4.43–4.65)	2.168 (0.016) ††	0.192	1.440 (0.924)	0.131	1.477 (0.225)	0.006
Availability of Foods for Child to Prepare for Breakfast	4.27 ± 0.86 (4.12–4.42)	4.47 ± 0.69 (4.35–4.59)	4.51 ± 0.72 (4.38–4.63)	4.46 ± 0.73 (4.33–4.58)	3.035 (0.0015)	0.254	0.717 (0.763)	0.072	1.773 (0.184)	0.007
Fruit/Vegetable Accessibility at Home [86] ^A^	4.06 ± 1.02 (3.88–4.23)	4.21 ± 0.86 (4.06–4.35)	4.20 ± 0.89 (4.05–4.35)	4.24 ± 0.84 (4.09–4.38)	1.879 (0.031) ††	0.158	0.511 (0.305)	0.047	0.096 (0.758)	<0.001
Fruit/Vegetable Accessibility in Neighborhood [86] ^A^	4.15 ± 0.73 (4.03–4.28)	4.26 ± 0.74 (4.14–4.39)	4.29 ± 0.70 (4.18–4.41)	4.28 ± 0.72 (4.16–4.40)	1.750 (0.041) ††	0.151	0.237 (0.593)	0.021	0.487 (0.486)	0.002
Physical Activity Environment [118] ^A^										
Indoor Space and Supports	4.03 ± 0.86 (3.89–4.18)	4.14 ± 0.80 (4.00–4.28)	4.22 ± 0.70 (4.10–4.34)	4.21 ± 0.84 (4.06–4.35)	1.405 (0.081)	0.129	0.192 (0.576)	0.019	0.016 (0.900)	<0.001
Outdoor/Yard Space and Supports †	4.51 ± 0.64 (4.39–4.63)	4.51 ± 0.71 (4.38–4.65)	4.60 ± 0.56 (4.50–4.70)	4.60 ± 0.60 (4.49–4.71)	0.066 (0.474)	0.007	0.086 (0.534)	0.007	0.195 (0.659)	0.001
Neighborhood Space and Supports	3.77 ± 0.96 (3.60–3.93)	3.83 ± 0.99 (3.66–4.00)	3.76 ± 1.01 (3.59–3.93)	3.90 ± 0.92 (3.74–4.06)	0.721 (0.236)	0.068	1.673 (0.048) ††	0.144	0.439 (0.508)	0.002
# Media Devices in Child’s Bedroom [86]	1.94 ± 2.00 (1.59–2.28)	1.76 ± 1.46 (1.51–2.02)	1.99 ± 1.80 (1.68–2.23)	1.86 ± 1.64 (1.58–2.14)	1.034 (0.152)	0.100	0.913 (0.181)	0.078	0.194 (0.660)	0.001

* Paired one-tailed *t*-tests in the expected direction (i.e., upper for all scores in the table, except the lower tail was used for Family Meals in Unhealthy Locations [days/week], Sugar-Sweetened Beverage Servings/Household Member/Day, and # Media Devices in Child’s Bedroom); effect size calculated with Cohen’s *d*. # Effect size was calculated using Cohen’s d for continuous variables. ## ANCOVA: Analysis of Covariance with baseline value as the covariate; effect size calculated with partial eta-squared (*ηp*^2^). ^A^ Five-point Agreement Rating, namely, strongly disagree, disagree, neutral, agree, and strongly agree, scored 1 to 5, respectively. All items in the scale are averaged to create the scale score, with higher scores indicating greater expression of the characteristic. † Experimental group n = 108 and control group n = 119, due to not having space for physical activity right outside their homes (yards) at baseline and/or post-survey. †† Benjamini–Hochberg procedures with a 5% or 10% false discovery rate indicate *P* is not significant.

## Data Availability

The final dataset is accessible only to project researchers.

## Trial Status

ClinicalTrials.gov, Protocol #NCT04802291, Registered 14 March 2021; Recruitment began in 2023 and concluded in 2024.
